# Syphilis screening in the antenatal care: a cross-sectional study from Botswana

**DOI:** 10.1186/1472-698X-6-8

**Published:** 2006-07-08

**Authors:** Maria Romoren, Mafizur Rahman

**Affiliations:** 1 Department of General Practice and Community Medicine, University of Oslo, Box 1130 Blindern, N-0318 Oslo, Norway; 2 Sexual and Reproductive Health Associates, Gaborone, Botswana

## Abstract

**Background:**

Congenital syphilis is recognized as a substantial public health problem in Sub-Saharan Africa. The aim of this study was to determine the prevalence of syphilis among antenatal care attendees in Botswana and to contribute to knowledge about the challenges facing the syphilis-screening programme.

**Methods:**

In a cross-sectional study, 703 antenatal care attendees at 13 health facilities in Gaborone, Botswana were interviewed and examined. Venous blood samples were collected for the identification of syphilis infection. The antenatal records were used to obtain information on any screening, diagnosis and treatment of syphilis that had been done earlier in the current pregnancy.

**Results:**

Active syphilis was found in 32 (5%) of the attendees. Among 546 women coming for a repeat antenatal care visit, 71 (13%) had not been screened for syphilis. Uptake late in pregnancy, delayed treatment and a high rate of seroconversion after testing were other identified obstacles to the effective prevention of congenital syphilis.

**Conclusion:**

Syphilis prevalence among pregnant women in Botswana remains high, and there is still much to be gained by improving the effectiveness of the syphilis screening and treatment programme. Earlier antenatal care attendance, rapid on-site testing, improved partner treatment and a repeat test late in pregnancy to manage incident cases are important goals for patients, health care workers and health authorities.

## Background

Maternal syphilis has a severe impact on pregnancy outcome. Although antenatal syphilis screening has proven to be cheap and effective, syphilis during pregnancy continues to be a substantial problem in resource-poor settings [1]. Published information from developing countries reveals that there is reason to improve both the coverage and the quality of the syphilis screening programme in antenatal care [2-4].

In Botswana, universal screening and treatment of syphilis in pregnancy is integrated in antenatal care. Blood is collected from all attendees at the first antenatal visit and analyzed with a non-treponemal syphilis test (the rapid plasma reagin [RPR] test or the venereal disease research laboratory [VDRL] test) at a centralized laboratory. According to the guidelines, high risk women should be retested at 34–36 weeks of gestation. All RPR/VDRL positive cases should be provided with education, risk reduction counselling and condoms. The treatment regimen is three doses of benzathine penicillin 2.4 million units at weekly intervals. Partner notification and treatment is not specifically recommended.

The striking need for improved surveillance of syphilis and its consequences in developing countries has been emphasized, [5] and this need is also evident in Botswana. Unfortunately, the test results in the screening programme are not routinely utilized to monitor the prevalence of syphilis among antenatal care attendees. There is also a lack of information on the proportion of pregnant women tested, the proportion who return for their results, treatment provided to test positive women and their partners and adverse birth outcomes due to syphilis.

The aim of this study was to determine the prevalence of syphilis among antenatal care attendees in Gaborone, Botswana; to contribute to increased knowledge of the implementation of the national syphilis screening programme; and to identify areas of concern in this and similar settings.

## Methods

Included in this study were 703 antenatal care attendees who visited 13 primary health care clinics in Gaborone, the capital of Botswana, between October 2000 and February 2001. Although 18 facilities in Gaborone provided antenatal care at that time, the 5 facilities with less than 600 antenatal care consultations during the previous year (range 10 to 570) were not included for practical reasons. A proportionate sample of attendees was recruited from each of the eligible clinics, based on the total load of antenatal care attendees at that clinic during the same period in the previous year. The clinics were visited one by one, and the attendees were consecutively invited to be included in the study. The number of patients per day was limited by either the total number of attendees at the clinic that day or by the number of specimens the laboratory could handle (which varied from 6 to 12, due to variation in staff and other activities at the lab). In the majority of clinics, all attendees were included during the period of data collection; in the busiest clinics only a random sample of the attendees was included.

All participants gave written, informed consent. The only exclusion criterion was the use of antibiotics in the two weeks prior to their visit. No woman declined to participate in the study, but 34 attendees were excluded – 32 because of treatment for vaginal discharge syndrome and 2 because they were on penicillin for syphilis.

A structured interview was used to obtain information on socio-demographic factors, symptoms of any sexually transmitted infection (STI) and history of STI diagnosis and treatment earlier in the pregnancy. All eligible attendees underwent a genital examination by a medical doctor, and clinical signs from external and internal genitalia were recorded. For the attendees coming for a repeat visit, we recorded the gestational age when blood was drawn for the non-treponemal syphilis test, the test results and the treatment prescribed, all which is documented in the patient-held antenatal records. For study purposes, additional venous blood samples were collected from both new and repeat attendees. The maternal sera were tested at the National Health Laboratory with the RPR test and the specific *Treponema pallidum *haemagglutination assay (TPHA).

Data were analyzed with the statistical package SPSS Version 11. The study was approved by the national committees for medical research ethics in Botswana and in Norway.

## Results

The median age of the 703 antenatal care attendees was 25 years (range 15–43). Other socio-demographic characteristics are described elsewhere [6]. Eight women reported symptoms and five women had signs of genital ulcers, but all of them were RPR negative. There were 157 women coming for the first antenatal visit, with a median gestational age of 20 weeks (range 8–37). Among the 546 women coming for a repeat visit, 71 (13%) had not been screened for syphilis in the routine antenatal programme. Among the 475 repeat attendees who had been tested, the median gestational age when the blood had been drawn was 19 weeks (range 4–38). Of these 475, 14 (3%) women had been found RPR positive during the screening. Two had not been treated; for the remaining 12 women, the mean time between blood being drawn and treatment being prescribed was five weeks. The screening test results for these 14 women, the treatment delay and the syphilis serology found in the study later in pregnancy is shown in Table 1. Among the women with negative RPR or those lacking RPR results earlier in pregnancy, of whom 103 were in gestational week >36, none had been retested.

**Table 1:** Routine syphilis screening and serological status in the study setting. Results among 14 antenatal attendees who were RPR positive in the screening programme.

**Screening programme**				**Study setting**		
Routine RPR result	Blood drawn gestational week	Treatment gestational week	Treatment prescribed	Gestational week	RPR result	TPHA

Reactive	20	24	Standard regimen*	34	1:32	Positive
Reactive	27	-	No treatment†	30	1:16	Positive
1:8	16	20	Standard regimen	32	1:16	Positive
1:16	23	25	Standard regimen twice	39	1:16	Positive
1:2	14	18	Standard regimen	39	1:8	Positive
Reactive	14	19	Single-dose penicillin	29	1:2	Positive
Reactive	20	24	Standard regimen	31	Non-reactive	Positive
1:2	19	-	No treatment	28	1:1	Negative
1:4	15	27	Standard regimen	38	Non-reactive	Negative
1:4	25	30	Standard regimen	36	Non-reactive	Negative
1:2	17	25	Single-dose penicillin	36	Non-reactive	Negative
1:2	14	19	Single-dose penicillin	40	Non-reactive	Negative
1:2	19	23	Standard regimen	33	Non-reactive	Negative
1:4	15	19	Standard regimen	38	Non-reactive	Missing

*Botswana guidelines for the management of RPR/VDRL positive cases  (regardless of titre): Three injections with benzathine penicillin 2.4  million units at weekly intervals.

†The patient came for the third  antenatal care visit.

RPR titre and TPHA results in the study setting are shown in Table 2. A total of 74 (11%) of the 703 attendees were TPHA positive, indicating past or present syphilis. In all, 32 (5%) women had active syphilis (RPR+/TPHA+) and 11 (2%) had high-titre active syphilis (RPR ≥ 1:8/TPHA+). Of the 9 repeat attendees identified in the study with high-titre active syphilis, 1 had not been tested in the routine screening, 3 had tested negative and 1 had tested positive but was not treated. The last 4 had been treated (mean of 10 weeks earlier), but still had laboratory-diagnosed high-titre syphilis, raising a concern about reinfection. Only 1 of the 17 repeat attendees identified in the study with low-titre active syphilis had been identified in the routine antenatal care.

**Table 2:** Syphilis prevalence among 692* antenatal care attendees in Gaborone, Botswana

	New attendees n (%)		Repeat attendees n (%)		All attendees n (%)	
*RPR positive cases*						
RPR titre ≥8, TPHA positive (HTS)	2	(1)	9	(2)	11	(2)
RPR titre <8, TPHA positive (LTS)	4	(3)	17	(3)	21	(3)
Biologically false positive†	2	(1)	12	(2)	14	(2)
*RPR negative cases*						
Past or treated syphilis‡	11	(7)	31	(6)	42	(6)
Uninfected	138	(88)	466	(87)	604	(87)

Total	157	(100)	535	(100)	692	(100)

RPR, rapid plasma reagin; TPHA, *Treponema pallidum *haemagglutination assay; HTS, high-titre syphilis; LTS, low-titre syphilis

*Among 546 repeat attendees, 11 were excluded from this analysis because the TPHA was missing.

† TPHA-negative

‡ TPHA-positive

We do not have data on the exact incidence of syphilis during pregnancy, but among the 461 attendees who were RPR negative in the routine screening, 451 were retested in the study setting with both RPR and TPHA. Median 11 weeks later in their pregnancy, 16 (3.5%) of these 451 initially RPR negative women had active syphilis; 1 with high and 15 with low titres. Of the 16 women, 15 reported having had one partner the last 12 months, and 14 said that they had been in the relationship more than two years.

## Discussion

Maternal syphilis remains a significant cause of adverse pregnancy outcome in Sub-Saharan Africa [2]. Antenatal syphilis screening is cost-effective even at RPR prevalences as low as 2% [7]. The syphilis prevalence in Botswana must still be considered high. In our study from Gaborone, 7% of the pregnant women were RPR positive. In the national HIV/AIDS surveillance in 2003, 5% of the antenatal care attendees were RPR positive. In the 2005 surveillance, 3% of the attendees were RPR positive [8,9]. Rural districts seem to be in special need of intensified prevention efforts, as prevalences up to 13% were found in less densely populated areas such as the Kalahari Desert. A recent review of laboratory logbooks in Francistown, the largest town of northern Botswana, found that the prevalence of VDRL positive antenatal care attendees declined from 12% in 1992 to 4% in 2003 [10]. The authors suggest that a decline in risky sexual behaviour due to interventions to control the HIV epidemic may have resulted in a reduced prevalence of syphilis. The introduction of syndromic management of STIs in 1992, which implies that 150000 to 200000 STI clients have been treated with multiple antibiotics yearly, may also have contributed to reduce the prevalences of curable STIs. It is critical that data showing a possible encouraging trend do not lead to reduced attention; rather, they should be used as a motivator for continued and improved syphilis control.

To prevent congenital syphilis, it is imperative to screen for syphilis early in pregnancy; but this remains a challenge. We found that the pregnant women who came for a first antenatal care visit had a median gestational age of 20 weeks. The repeat attendees had been tested at 19 weeks. Promotion of earlier attendance at antenatal care is a simple prevention strategy which should be advocated. The current practice of processing tests at centralized laboratories also results in delayed treatment; as described, it is takes more than a month before treatment is provided. To ensure immediate treatment, rapid diagnosis of syphilis can be introduced in the clinics, as recommended by the World Health Organization [11]. Botswana is in a favourable position to explore the use of such tests. Through the prevention of mother-to-child transmission of HIV (PMTCT) programme, all health posts and clinics have a lay counsellor with four weeks of training who performs rapid tests for HIV. Several authors have emphasized the need to identify synergies between the PMTCT and the syphilis screening programmes [5,12]. To utilize lay counsellors to perform simple, rapid point-of-care tests for both syphilis and HIV could prove a concrete and feasible opportunity to reduce the disease burden of these two infections.

This study from Botswana also identifies several other operational difficulties within the syphilis screening programme. Of the 26 repeat attendees who were identified with active syphilis (RPR and TPHA positive) in our study, only five had been identified and treated with penicillin in routine antenatal care. Not all antenatal care attendees had been tested, and not all attendees with positive RPR results had been treated. None of the attendees in the study population had been routinely tested more than once. As many as 3.5% of the women who were RPR negative in the routine screening were RPR and TPHA positive when re-tested in the study after a median period of 11 weeks. All but one of these women was considered to be at low risk of infection. The women's initial TPHA status is not known, and some of them could have had old or untreated syphilis combined with an RPR seroconversion due to conditions other than syphilis. Nevertheless, the results indicate a high number of incident cases, and we recommend an urgent change in the retesting policy. A repeat test late in pregnancy for both low- and high-risk attendees should be clearly stated in the guidelines and communicated to the health workers.

In the national STI management guidelines, partner notification and treatment is explicitly recommended for other STI syndromes, but not for RPR/VDRL positive cases. Botswana, like many other countries, relies on patient-based partner referral, with index patients informing their own contacts and referring them for treatment. To avoid reinfection of the patients and reduce the spread of syphilis, partners of RPR/VDRL positive antenatal care attendees should be treated. A patient-based contact tracing strategy should be the minimum strategy employed in the syphilis screening programme, and more intense efforts should be considered. It is known that partner notification and treatment can be improved by introducing combinations of provider and patient referral, and by explicit and thorough verbal health education [13].

Our results indicate a need for improved coverage of the screening and improved adherence to treatment guidelines. However, it is a limitation of the study that we do not have information on why the guidelines are not followed. Data on the impact of maternal syphilis on pregnancy outcome are also lacking. A targeted approach to improving the syphilis screening programme requires that more knowledge be gathered about the specific problems, where they exist, and why. There is an obvious need to strengthen the routine data collection. In order to evaluate the screening programme continuously, each clinic can easily register and report the proportion of pregnant women who are tested and the proportion of test-positive women who are effectively treated. Studies on the quality of STI care in Botswana have shown that the clinics have adequate supplies of equipment and drugs, but that nurses' adherence to the guidelines is poor [14,15]. Informing the clinics about the prevalence of syphilis and the screening and treatment coverage, together with in-service training and supervision, could increase the health care providers' commitment to the programme.

The study population is representative for antenatal care attendees in Gaborone, and the level of health care is supposed to be the same throughout the country. However, conditions such as communication, transport, educational level and standard of living are less favourable in the rural areas. To the best of our understanding, it is reasonable to expect the same or higher prevalence of syphilis, as well as the same or greater operational difficulties in the rest of the country. We also believe that the results of this study are of relevance to other developing countries.

## Conclusion

Syphilis remains a significant public health problem in many resource-poor settings. Lack of adequate antenatal care has been described as an important obstacle to the prevention of congenital syphilis globally, and available data show that this is also the case in Botswana. The prevalence of syphilis among pregnant women is high, and there is much to be gained by improving the effectiveness of the syphilis screening and treatment programme. We recommend promotion of earlier antenatal care attendance, rapid on-site testing, improved partner treatment and a repeat test late in pregnancy in order to manage incident cases. Increased commitment among both politicians and health care workers is necessary to reduce maternal syphilis and its consequences.

## Abbreviations

RPR, rapid plasma regain; TPHA, treponema pallidum hemaglutin assay; STI, sexually transmitted infection; PMTCT, the prevention of mother-to-child transmission of HIV programme

## Competing interests

The author(s) declare that they have no competing interests.

## Authors' contributions

Maria Romoren contributed to the study design, was responsible for data collection and data analysis, and was the primary author of the manuscript. Mafizur Rahman contributed to the study design and to formal and organisational aspects of the study and approved the final manuscript.

## Pre-publication history

The pre-publication history for this paper can be accessed here:


